# Transport and Co-Transport of Carboxylate Ions and Ethanol in Anion Exchange Membranes

**DOI:** 10.3390/polym13172885

**Published:** 2021-08-27

**Authors:** Jung Min Kim, Yi-hung Lin, Brock Hunter, Bryan S. Beckingham

**Affiliations:** Department of Chemical Engineering, Auburn University, Auburn, AL 36849, USA; jzk0090@auburn.edu (J.M.K.); yzl0260@auburn.edu (Y.-h.L.); bjh0062@auburn.edu (B.H.)

**Keywords:** charge screening, competitive diffusion, multi-component transport, in situ ATR–FTIR spectroscopy, anion exchange membrane

## Abstract

Understanding multi-component transport behavior through hydrated dense membranes is of interest for numerous applications. For the particular case of photoelectrochemical CO_2_ reduction cells, it is important to understand the multi-component transport behavior of CO_2_ electrochemical reduction products including mobile formate, acetate and ethanol in the ion exchange membranes as one role of the membrane in these devices is to minimize the permeation of these products. Anion exchange membranes (AEM) have been employed in these and other electrochemical devices as they act to facilitate the transport of common electrolytes (i.e., bicarbonates). However, as they act to facilitate the transport of carboxylates as well, thereby reducing the overall performance, the design of new AEMs is necessary to improve device performance through the selective transport of the desired ion(s) or electrolyte(s). Here, we investigate the transport behavior of formate and acetate and their co-transport with ethanol in two types of AEMs: (1) a crosslinked AEM prepared by free-radical copolymerization of a monomer with a quaternary ammonium (QA) group and a crosslinker, and (2) Selemion^®^ AMVN. We observe a decrease in diffusivities to carboxylates in co-diffusion. We attribute this behavior to charge screening by the co-diffusing alcohol, which reduces the electrostatic attraction between QAs and carboxylates.

## 1. Introduction

**Figure 1:**
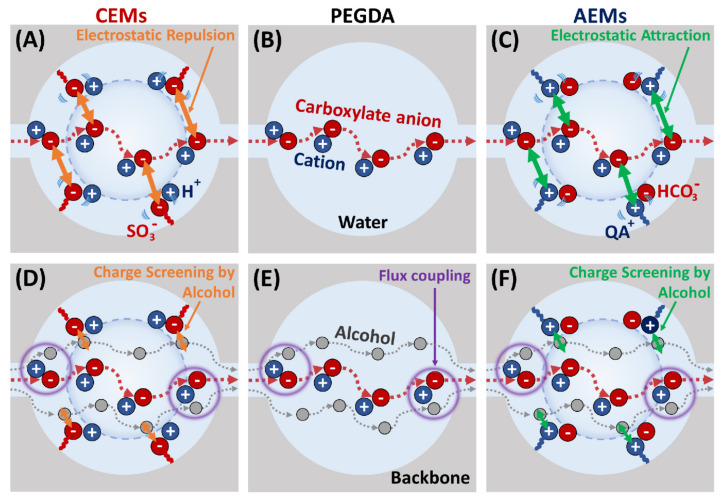
Schematic depiction of carboxylate salt diffusion in (**A**,**D**) cation exchange membranes (i.e., PEGDA-AMPS and Nafion^®^ 117), (**B**,**E**) crosslinked PEGDA (i.e., A0) and (**C**,**F**) anion exchange membranes (i.e., P8, P12 and AMVN) in (**A**–**C**) single and (**D**–**F**) co-diffusion with an alcohol (MeOH or EtOH). Figures are reprinted in part from [28,30,32] with permission from Elsevier and Wiley.

Anion exchange membranes (AEM [1,2]) are a crucial component of devices for various applications, including direct ethanol fuel cells [3], direct urea fuel cells [4], water purification devices [5], water electrolyzers [6], CO_2_ electrolyzers [7] and photoelectrochemical CO_2_ reduction cells (PEC-CRC) [8,9,10,11,12]. Of particular interest here are PEC-CRCs, which utilize solar power to reduce CO_2_ to various chemicals [13,14], such as methanol (MeOH), ethanol (EtOH), formate (OFm^−^) and acetate (OAc^−^). Major roles of the AEM in such devices are to provide preferential ion transport (i.e., hydroxide (OH^−^ and anionic electrolytes (bicarbonates, HCO_3_^−^) [15,16]) with membrane-bound, charged functional groups (i.e., quaternary ammonium (QA^+^) for AEMs) and to minimize the permeation of CO_2_ reduction products to the anode chamber, as they readily oxidize back to CO_2_ and by-products [11,12]. While the majority of ion exchange membranes (IEM [17,18,19,20]) designed for PEC-CRCs have focused on AEMs, one of the major drawbacks of AEMs for such devices is their high diffusibility for negatively charged CO_2_ reduction products (OFm^−^ and OAc^−^). To avoid this issue, our group has performed a series of investigations to gain a fundamental understanding of cation exchange membranes (CEM [21,22,23,24]) for PEC-CRCs, such as how the presence of a series of charge-neutral comonomers (acrylic acid, hydroxyethyl methacrylate and poly(ethylene glycol) methacrylate (PEGMA) [25,26]) or how the presence of co-diffusing neutral CO_2_ reduction products (alcohols) can act to impact and, in some cases, mitigate the permeation of CO_2_ reduction products (carboxylates) [27,28,29,30,31,32]); see Figure 1A,D. In the case of Nafion^®^ 117 (a commercial CEM) and PEGDA-AMPS (a crosslinked CEM that we prepared by incorporating 2-acrylamido-2-methyl-1-propanesulfonic acid (AMPS, sulfonate-containing ionomer) with a crosslinker, poly(ethylene glycol) diacrylate (PEGDA)), both OFm^−^ and OAc^−^ diffusivities were increased in co-diffusion with either MeOH or EtOH, where we conjectured a potential charge screening by co-diffusing alcohol [32]. In the case of PEGDA-AMPS CEMs, which also incorporated a charge-neutral comonomer (PEGDA-AMPS/AA/HEMA, and/PEGMA) into the structure, the OAc^−^ diffusivities in PEGDA-AMPS/PEGMA and/HEMA were increased (as in comonomer-free PEGDA-AMPS films) [28]. However, those of PEGDA-AMPS/PEGMA were the same [28] or slightly decreased in co-diffusion with MeOH, where we conjectured a potential charge screening by long pendant chains.

**Figure 2:**
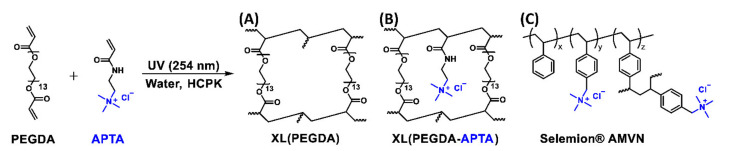
(**A**,**B**) Synthetic scheme of (**A**) crosslinked PEGDA, A0, and (**B**) crosslinked PEGDA-APTA, A8 and A12. (**C**) Schematic of Selemion AMVN, functionalized polystyrene-divinylbenzene (PS-DVB)-based film.

Here, to further investigate multicomponent transport behavior [33] in IEMs, we perform an analogous investigation on a series of AEMs, Selemion^®^ AMVN (AMVN) and PEGDA-APTA (A8 and A12). AMVN is a commercial AEM and PEGDA-APTA is a crosslinked AEM that we prepare by incorporating (3-acrylamidopropyl) trimethylammonium chloride (APTA, QA^+^-containing ionomer) with a crosslinker, PEGDA [28,30,34,35]; see Figure 2.

Moreover, we prepare and characterize a crosslinked PEGDA (A0) as an uncharged analog to exclude the effect of QA^+^ from A8 and A12 for comparison. The aim of this work is to examine how the presence of co-permeating EtOH impacts the transport behavior and whether this behavior is consistent with our prior findings for similar systems. Previously, Carter et al. investigated the co-transport of alcohols (MeOH, EtOH and n-PrOH) in Selemion AMV, where they observed a competitive sorption and flux coupling behavior in co-transport [8]. Based on our prior work described above on the co-transport of carboxylates and alcohols in CEMs, a pictorial description of how the presence of co-permeating alcohols could be interfering with the electrostatic interactions (repulsion for CEMs and attraction for AEMs [18,36,37]) between membrane-bound charge groups (sulfonates for CEMs or QA^+^ for AEMs) and a mobile carboxylate anion is shown in Figure 1. In Figure 1A–C, the diffusion of the carboxylate anion by itself is depicted, where the mobile carboxylate anion experiences electrostatic repulsion from bound sulfonate in CEMs (Figure 1A) and electrostatic attraction from bound QA^+^ in AEMs (Figure 1C) (ion-polymer interaction). In Figure 1D–F, the diffusion of carboxylates is assisted by co-diffusing alcohols (flux coupling; [38]) (ion–alcohol interaction), where the electrostatic interaction between bound charge groups and the mobile carboxylate anion is screened by the co-permeating alcohol (alcohol–polymer interaction, charge screening; [27,29,30,31]; Figure 1D,F).

To probe this behavior in the AEMs of interest here, for each AEM, we measure their permeability (*P_i_*) and solubility (*K_i_*) to both K^+^ and Na^+^ forms of formate (OFm^−^) and acetate (OAc^−^). Permeabilities are measured by diffusion cell experiments coupled with in situ attenuated total reflectance–Fourier transform infrared (ATR–FTIR) spectroscopy [29]; solubilities are measured by sorption–desorption experiments coupled with high-performance liquid chromatography (HPLC) [22]. Additionally, we measure carboxylate permeability (in co-diffusion) and solubility (from a mixture) with ethanol (EtOH). We then calculate diffusivities (*D_i_*) to OFm^−^ and OAc^−^ in both K^+^ and Na^+^ forms (KOFm, KOAc, NaOFm and NaOAc) using the solution-diffusion model (Equation (1)) [39], which describes the overall solute permeation, which is dependent on solute sorption [22,40,41,42,43] into the membrane and diffusion [18,42,44,45] through the fractional free volume [46,47,48,49] within the polymer matrix,

*P*_*i*_ = *D*_*i*_*K*_*i*_, (1)

where $P_{i}$ is the permeability to solute *i*, $D_{i}$ is the diffusivity to solute *i*, and $K_{i}$ is the solubility to solute *i*, for EtOH [8,50] or a carboxylate anion (OFm^−^ or OAc^−^ [51,52]) in single and co-transport between EtOH and a carboxylate (EtOH-KOFm, EtOH-KOAc, EtOH-NaOFm and EtOH-NaOAc. The multi-solute permeability measurement was enabled by our in situ ATR–FTIR probe approach, which we recently devised [29]. This method is particularly useful upon measuring the alcohol permeabilities and multi-solute permeabilities as the alternative methods typically involve aliquots, where the loss of the solution in either the feed or receiver cell is inevitable and characterization of the multicomponent aliquots ex situ can be a challenge (especially for volatile species). Ultimately, we analyze and discuss the observed multi-solute transport behavior in these AEMs, which will allow more target-specific design of membranes for CO_2_ reduction cells.

## 2. Materials and Methods

### 2.1. Materials

Potassium formate (KOFm, ≥98%) was purchased from BeanTown Chemical (Hudson, NH, USA). Potassium acetate (KOAc, ≥99.0%), potassium chromate (K_2_CrO_4_, 5% (*w*/*v*)) and silver nitrate (AgNO_3_, 1 M) were purchased from British Drug Houses (Poole, UK). Sodium formate (NaOFm, ≥99%) was purchased from Alfa Aesar (Haverhill, MA, USA). Sodium acetate (NaOAc, ≥99%) was purchased from ACS Chemical Inc. (Point Pleasant, NJ, USA). Ethanol (EtOH, ≥99%), poly(ethylene glycol) diacrylate (PEGDA, *n* = 13) and (3-acrylamidopropyl) trimethylammonium chloride solution (APTA, 75 wt.% in water) were purchased from Sigma-Aldrich Chemicals (St. Louis, MS, USA). Moreover, 1-Hydroxyl-cyclohexyl phenyl ketone (HCPK, photoinitiator) was purchased from Tokyo Chemical Industry (Tokyo, Japan). Selemion^®^ AMVN (AMVN) was purchased from AGC Engineering Co. (Tokyo, Japan); see Figure 2C for the schematic. All water used in this investigation was Type-1 deionized water produced by a Waterpro BT Purification System from Labconco^®^ (18.2 mΩ·cm at 25 °C, 1.2 ppb TOC) (Kansas City, MO, USA).

### 2.2. PEGDA and PEGDA-APTA Membranes

The detailed procedure on film formation is described in previous work [28,30,35]. A PEGDA membrane and two PEGDA-APTA anion exchange membrane (AEM) were prepared by UV photopolymerization of prepolymerization mixtures, as shown in Figure 2A,B and Table 1. In the film name (A#), A represents APTA and # represents the mol% of the APTA content. For example, A12 denotes the film prepared with PEGDA (88 mol%) and APTA (12 mol%). Each pre-polymerization mixture was prepared with 20 wt.% of water content and HCPK (0.1 wt.% of the polymer) (Table 1); see Supporting Information Figure S1 for photographs. Essentially complete conversion was achieved as the mass of polymer network-forming monomers in the prepolymerization mixtures was in line with the mass of the films after vacuum drying at 50 °C following 5 days of swelling in DI water within ~99% [26,53].

### 2.3. Counterion Conversion via Ion Exchange from Cl^−^ to HCO_3_^−^

The chloride (Cl^−^) counterion in all AEMs (A8, A12 and AMVN) was exchanged to bicarbonate (HCO_3_^−^). Then, 0.75-inch and 1-inch hole-punches (General Tools 1271 Arch Punches) were used to cut each AEM into 0.75-inch films for sorption–desorption experiments and 1-inch films for diffusion cell experiments. All films were then placed in 1 M sodium bicarbonate (NaHCO_3_) for 2 days, where the solution was replaced daily and gently stirred [16]. Next, all films were washed with DI water and placed in DI water for 2 days to remove excess Cl^−^ and NaHCO_3_, where the solution was gently stirred, and water was replaced daily. A conductivity meter was used to confirm that the conductivity of the solution was the same as DI water (≤18.2 mΩ·cm at 25 °C). The degree of conversion for all films from Cl^−^ to HCO_3_^−^ was measured by elemental analysis using a scanning electron microscope (Zeiss EVO 50 SEM) coupled with energy dispersive spectroscopy (INCA EDS).

### 2.4. Ionic Conductivity Measurement

In-plane conductivity of all films was measured using a four-point conductivity cell (BekkTech BT-110) employed with a Gamry Interface 1000 potentiostat [30]. A rectangular section of the film (length: >1.0 cm, width (*W*): 0.5 cm) was cut and placed in the conductivity cell. The cell was then placed in DI water (500 mL), and electrochemical impedance spectroscopy (EIS) was performed after stabilization of the open circuit potential (frequency: 10 Hz–1 MHz, AC voltage: 10 mV). The EIS data were analyzed in Gamry Echem Analyst software and the resistance, *R* (Ω), obtained from the Nyquist plot. The ionic conductivity, *σ*, was measured as follows:

(2) $$\sigma =\frac{L}{RWT}$$

where *L*, *W* and *T* are the distance between two electrodes (0.5 cm), the width and the thickness of the film, respectively.

### 2.5. Ion Exchange Capacity

Ion exchange capacity (IEC, mmol/g) was measured using the Mohr method [54,55,56] for A8 and A12. Briefly, all hydrated membranes were dried in a vacuum oven at 50 °C for 24 h. The mass of the dried films (~1.5 g), $W_{d}$, was measured. Each film was placed in 1 M NaOH (~50 mL) for more than 2 days (to replace Cl^−^ with OH^−^). Each solution was then poured into a beaker filled with ~150 mL of water and ~5 mL of the K_2_CrO_4_ solution (5% in water). Lastly, 0.1 M AgNO_3_ solution was added dropwise until the color of the solution remained red-brown. The IEC was calculated as follows:

(3) $$IEC=\frac{V_{AgNO_{3}}\times C_{AgNO_{3}}}{W_{d}}$$

where $V_{AgNO_{3}}$ is the volume of AgNO_3_ solution added, $C_{AgNO_{3}}$ is the concentration of the AgNO_3_ solution (0.1 M), and $W_{d}$ is the mass of the dried film.

### 2.6. Water Content, Density and Water Volume Fraction

Water uptake was measured gravimetrically. A 0.75-inch diameter hole-punch was used to cut each hydrated film. The mass of each hydrated film, $W_{s}$, was measured after quickly blotting them with tissue paper. The films were then dried under a vacuum at 50 °C for 24 h and the mass of each dried film, $W_{d}$, measured [29]. The water uptake, $\omega_{w}$, was calculated as follows:

(4) $$\omega_{w}=\frac{W_{s}-W_{d}}{W_{d}}\times 100\%$$

where $W_{s}$ is the mass of the swollen film and $W_{d}$ is the mass of the dried film. 

Film density was measured by the buoyancy method with a density kit (ML-DNY-43, Mettler Toledo) coupled with a scale (ML204T, Mettler Toledo) [48]. The density, *ρ_p_*, was calculated as follows:

(5) $$\rho_{p}=\left(\rho_{L}-\rho_{0}\right)\left(\frac{W_{0}}{W_{0}-W_{L}}\right)+\rho_{0}$$

where *ρ_L_* is the density of water (997.8 kg/m^3^ at 22 °C), *ρ*_0_ is the density of air (1.225 kg/m^3^), *W*_0_ is the weight of the dried film in air, and *W_L_* is the weight of the film in water.

Water volume fraction, *ϕ_w_*, was calculated as follows:

(6) $$\phi_{w}=\frac{\left(W_{s}-W_{d}\right)/\rho_{p}}{\left(W_{s}-W_{d}\right)/\rho_{L}+W_{d}/\rho_{p}}$$

### 2.7. Diffusion Cell Experiment

A more detailed experimental method is thoroughly discussed elsewhere [8,29]. Briefly, permeabilities of A0, A8, A12 and AMVN to EtOH and carboxylate salts (KOFm, KOAc, NaOFm and NaOAc) were measured using a temperature-jacketed custom-built diffusion cell coupled with an in situ ATR–FTIR probe (Mettler-Toledo ReactIR™ 15 with a shallow tip 9.5 mm DSun AgX DiComp probe) for EtOH-containing solutions (single solute EtOH and EtOH-carboxylate salts) and a conductivity probe (PC820 Precision Benchtop, Apera Instruments, Schaumburg, IL, USA) for EtOH-free solutions (single solute carboxylate salts) to detect the evolving solute concentration in the receiver cell at 25 °C. The feed cell was initially filled with either a unary solution (1 M EtOH, KOFm, KOAc, NaOFm or NaOAc) or a binary solution (1 M each EtOH-KOFm, EtOH-KOAc, EtOH-NaOFm and EtOH-NaOAc), while the receiver cell was initially filled with DI water. The time-resolved concentrations of each solute were measured from the time-resolved absorbances acquired from the solution in the receiver cell and fitted to Yasuda’s model to calculate the permeability [57,58]. The osmotic flow of water from the receiver cell to the feed cell is neglected in this study as the difference due to osmotic flow was within the experimental error for 1 M methanol, NaOFm and NaOAc in a commercial ion exchange membrane (Nafion^®^ 117) [29].

### 2.8. Sorption–Desorption Experiment

Solubilities of A0, A8, A12 and AMVN to EtOH and carboxylate salts (KOFm, KOAc, NaOFm and NaOAc) were measured by a sorption–desorption technique in 5 unary solutions (1 M EtOH, KOFm, KOAc, NaOFm and NaOAc) and 4 binary solutions (EtOH-a salt) [22]. The 0.75-inch hole-punch was used to cut the PEGDA films (other AEMs were cut during the counterion conversion). Each film was quickly blotted with tissue paper and immersed in a solution vial (15 mL), either a unary solution or a binary solution, and each solution was prepared in triplicate. All films were placed in the solution vials for 2 days, where the solution was replaced daily. A digital caliper (±1 μm) was used to measure the film thickness by finding an average of five random locations, and ImageJ software (National Institutes of Health, Bethesda, MD, USA) was used to calculate the area of the films from digital photographs. Each film was then quickly blotted dry and immersed in a vial of DI water (10 g) for 2 days. The solution from each vial was then transferred to a high-performance liquid chromatography (HPLC) device, a refractive index detector with a Aminex HPX-87H column (Bio-Rad, Shanghai, China), to determine the solute concentration in each desorption solution. The solubility, $K_{i}$, of the solute *i* was calculated as:

(7) $$K_{i}=\frac{C_{i}^{m}}{C_{i}^{s}}$$

where $C_{i}^{m}$ is the concentration of the solute *i* in the film, which is the product of the concentration of the solute *i* of the desorption solution and the volume of the desorption solution (10 mL) divided by the volume of solution-soaked films, and $C_{i}^{s}$ is the concentration of the solute *i* in the external solution (1 M). The effect of condensed carboxylate anions (OFm^−^ or OAc^−^) to QA^+^ groups is neglected in this study [22], as (1) the solubilities can be adjusted with the IEC (as a complete conversion is expected from the EDS elemental analysis) and (2) we assumed that the effect of condensed carboxylates on co-diffusion was relatively small compared to the interaction between EtOH and uncondensed carboxylate. 

Total volume of the solution in films after sorption was calculated by subtracting the dry volume of the films (dry polymer mass/dry polymer density, *ρ_p_*) from the swollen volume of the films. Volume of each solute *i* in swollen films, $V_{i}$, was calculated as:

(8) $$V_{i}=\frac{n_{i}\times M_{i}}{\rho_{i}}$$

where $n_{i}$ is the mol of solute *i* (from the desorption solution), $M_{i}$ is the molecular mass of solute *i*, and $\rho_{i}$ is the density of solute *i*.

### 2.9. Diffusivity Calculation and Estimation

Diffusivities of all films to a solute in single diffusion and in co-diffusion (EtOH and a salt) were calculated using the solution-diffusion relationship (Equation (1)) [29,42,59]. The calculated diffusivities were then compared to the Mackie–Meares model [60]:

(9) $$D_{i}=D_{0,i}{\left(\frac{\phi_{w}}{2-\phi_{w}}\right)}^{2}$$

where *D_i_* is the diffusivity of a membrane to a solute *i* and *D*_0*,i*_ is the solute diffusivity in pure water; see Supporting Information Table S1 for values.

## 3. Results

**Figure 3:**
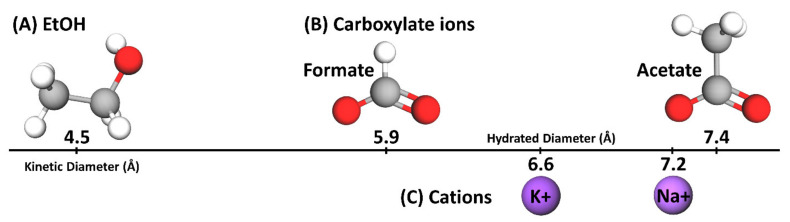
Molecular structure of (**A**) EtOH (4.5 Å), (**B**) carboxylate ions, OFm^−^ (5.9 Å) and OAc^−^ (7.4 Å) and (**C**) cations, K^+^ (6.6 Å) and Na^+^ (7.2 Å), where kinetic diameters are stated for EtOH and hydrated diameters are stated for ions. Carbons are shown in grey, oxygens are shown in red, hydrogens are shown in white, K^+^ is shown in a darker purple, and Na^+^ is shown in a lighter purple.

A charge-neutral film (A0) and three positively charged AEMs (A8, A12 and AMVN) were prepared to investigate the effect of polymer-bound quaternary ammonium cations (QA^+^) on OFm^−^-containing salts (KOFm and NaOFm) and OAc^−^-containing salts (KOAc and NaOAc) in single and co-transport with EtOH. To further understand this behavior, the EtOH transport of all films in single and co-transport with each salt was also analyzed. The permeabilities, solubilities and diffusivities of each solute in all films were measured; see Supporting Information Tables S2–S4 for values. These values were then analyzed based on three parameters: (1) the charge densities of cations, Na^+^ (0.14 mC/cm^2^) > K^+^ (0.07 mC/cm^2^) [61], (2) the hydrated diameters of cations, K^+^ (6.6 Å [62]) < Na^+^ (7.2 Å [62]) and anions, OFm^−^ (5.9 Å [51]) < OAc^−^ (7.4 Å [51]), and the kinetic diameter of EtOH (4.5 Å [62]) (Figure 3), and (3) the in-water diffusivities of cations, K^+^ (2.0 × 10^5^ cm^2^/s [63]) > Na^+^ (1.3 × 10^5^ cm^2^/s [63]), anions, OFm^−^ (1.5 × 10^5^ cm^2^/s [64]) > OAc^−^ (1.1 × 10^5^ cm^2^/s [63,64]) and EtOH (1.23 × 10^5^ cm^2^/s [65]). The relative kinetic diameters and hydrated diameters are shown in Figure 3.

### 3.1. Water Uptake, Density and Water Volume Fraction

Water uptakes, dry polymer densities and water volume fractions of films were measured gravimetrically, with results shown in Table 2. Generally, the water uptakes of PEGDA-based films (A0, A8 and A12) were higher than that of the PS-DVB-based film (AMVN, see Figure 2 for structure) by four times, on average. This is due to differences in the polymer backbones, where PEGDA-based films consist of a hydrophilic backbone (PEG) and AMVN consists of a hydrophobic backbone (PS-DVB). Water uptakes of A8 and A12 were higher than A0 by 3 and 6%, respectively, for those in Cl^−^ form and by 13 and 22%, respectively, for those in HCO_3_^−^ form. This is likely due to the increase in the free volume in films with decreasing crosslink density (PEGDA content) and increasing charged quaternary ammonium (QA^+^) content (APTA content) [35]. Generally, water uptakes of AEMs (A8, A12 and AMVN) in HCO_3_^−^ form were higher than those in Cl^−^ form by 15%, on average, analogous to prior results for QA^+^-poly(sulfone)-based AEMs reported elsewhere [15]. Here, the hydration number plays a role as the hydration number of HCO_3_^−^ (7–8 [66]) is higher than that of Cl^−^ (5.1–8.4 [67,68]) and, therefore, the films in HCO_3_^−^ form are more likely to hold more water molecules compared to the films in Cl^−^ form.

The dry polymer density of A0 (1.22 g/mL) is consistent with previously reported values [35,41,48]. Generally, the densities of the PEGDA-based films are higher than those of PS-DVB-based AMVN, in part due to the difference in atomic compositions; see Supporting Information Table S5 for values. For instance, PEGDA-based films contain ~35% of oxygen (16 g/mol) and ~65% of carbon (12 g/mol), whereas AMVN contains only ~5% of oxygen and ~94% of carbon. Moreover, the densities of AEMs in HCO_3_^−^ form are slightly higher than those in Cl^−^-form, which is attributed to the higher density of HCO_3_^−^ compared to Cl^−^ (i.e., the densities of KHCO_3_ and KCl are 2.17 and 1.98 g/mL, respectively).

The diffusivity of a solute in a hydrated, dense membrane is often described by free volume theory, in which solute diffusion occurs through the vacant and transient space between repeating polymer chains [57,58]. To describe this behavior, Yasuda et al. assumed that all the fractional free volume (FFV) within a hydrated film would be filled with water and proposed the following equation:

(10) $$D_{i}=D_{0,i}\exp\left[-\left(\frac{1}{\phi_{w}}-1\right)\right]$$

where *D_i_* is the diffusivity of a membrane to a solute *i*, *D*_0*,i*_ is the solute diffusivity in pure water, *B* is the empirical constant for each polymeric material, and *ϕ_w_* is the water volume fraction. Therefore, Equation (9) predicts solute diffusivity to rapidly increase with the water volume fraction and gradually equilibriate toward the solute diffusivity in pure water (*ϕ_w_* = 1). Assuming that the empirical constants do not differ drastically, the solute diffusivities of PEGDA-based films (A0, A8 and A12) will be higher than those of AMVN due to the differences in water volume fraction; see Table 2. We will return to this point in our discussion of solute diffusivities calculated using the solution-diffusion equation below.

### 3.2. Counterion Conversion, Ionic Conductivity and IEC

The weight compositions of carbon, oxygen and chloride were measured from EDS elemental analysis on A8, A12 and AMVN before and after the counterion conversion; see Supporting Information Table S5 for values. Generally, the carbon and oxygen compositions of both A8 and A12 were closely matched with the theoretical compositions from the pre-polymerization mixture. However, chlorine compositions were lower than the theoretical values by three times, on average. Complete counterion conversion (Cl^−^ to HCO_3_^−^) is presumed in all AEMs (A8, A12 and AMVN) as Cl^−^ was not detected in EDS elemental analysis on any film after the conversion [16]; see Figure S2.

The ionic conductivity (σ) and ion exchange capacity (IEC) of each AEM (A8, A12 and AMVN) were determined, yielding the results shown in Table 3. Generally, the measured IECs for both A8 and A12 are close to that of the theoretical IEC (calculated from the composition of the pre-polymerization mixture). This indicates that essentially complete conversion from monomers (PEGDA and APTA) to a crosslinked film has been achieved. The IEC of AMVN (1.5 meq/g) was significantly higher than those of PEGDA-based films by an order of magnitude, such that considerably more interactions between bound quaternary ammonium groups (QA^+^) and mobile species (K^+^, Na^+^, OFm^−^, OAc^−^ and EtOH) are expected for AMVN. Consequently, the ionic conductivity of AMVN is also greater than A8 and A12, by six times, on average.

**Figure 4:**
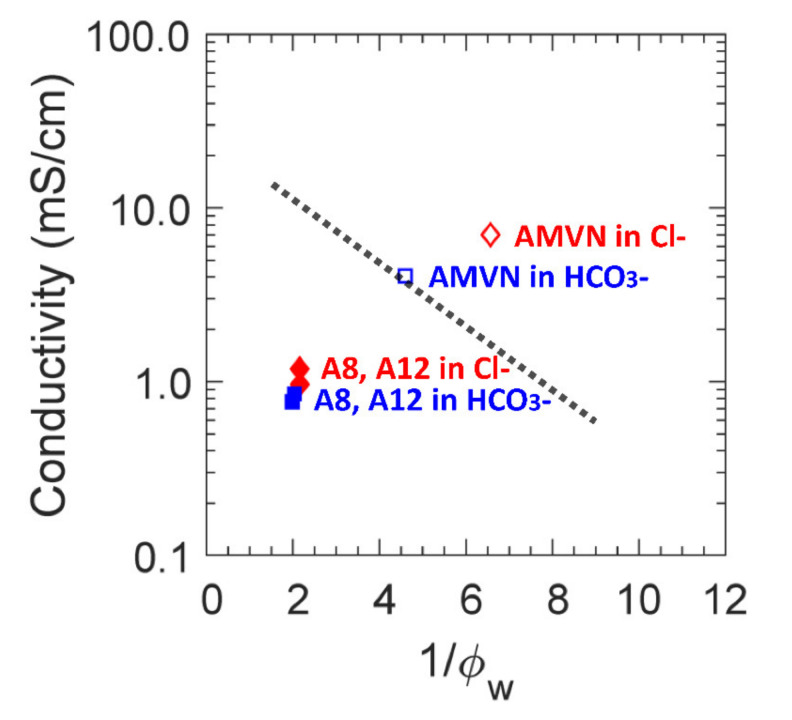
Ionic conductivity as a function of inverse water volume fraction for A8, A12 (filled markers) and AMVN (empty markers) in Cl^−^ (diamonds, ◊) and in HCO_3_^−^ (squares, □). The line is a regression on a series of ImPPO-χ AEMs and Selemion^®^ AMV from the literature [16].

The ionic conductivities of the AEMs in HCO_3_^−^ form were lower than those in Cl^−^ form by 1.4 times, on average [16]. The ionic conductivities of films in both Cl^−^ and HCO_3_^−^ form are plotted in a function of inverse water volume in Figure 4 along with the upper bound regression line for a series of Selemion^®^ AMV and ImPPO-χ AEMs [16]. While the conductivities of AMVN are within the range of other AEMs (Selemion^®^ AMV and ImPPO-χ [16]), the conductivities of both A8 and A12 are lower than their expected conductivity for their respective water volume fractions. This indicates that the transport behavior in A8 and A12 is expected to be closer to that of hydrated, dense membranes (i.e., A0) over the state-of-the-art AEMs (i.e., AMVN, Selemion^®^ AMV and ImPPO-χ).

### 3.3. Permeation

**Figure 5:**
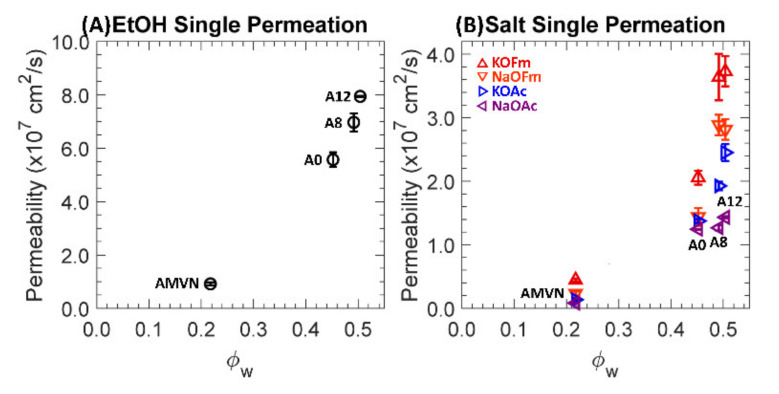
(**A**) Permeabilities to EtOH, ○, in single permeation. (**B**) Permeabilities to KOFm (△, red), NaOFm (▽, orange), KOAc (▷, blue) and NaOAc (◁, purple) in single permeation. Each data point is the average of 3 experiments, with error bars corresponding to the standard deviation.

One-component permeabilities to EtOH and carboxylate salts (KOFm, NaOFm, KOAc and NaOAc) of a charge-neutral A0 and positively charged A8, A12 and AMVN films in HCO_3_^−^ form are shown in Figure 5, where (A) and (B) are scaled differently. Generally, the thickness of AMVN films after permeation was essentially the same (all within 5%) and those of PEGDA-based films were slightly decreased (7–17%) with increasing APTA content; see Supporting Information Table S6. Permeabilities across all films were increased with increasing water volume fraction, showing similar results to those reported elsewhere [16,30,42,48]. This is primarily due to increased diffusion, where solute diffusivities tend to increase with increasing water volume fraction (i.e., free volume theory [57,58]); see Supporting Information Table S2 for values. For all films, salt permeabilities are in the order of KOFm > NaOFm > KOAc > NaOAc, indicating that the primary discrimination is the size difference between the two carboxylate anions, OFm^−^ (5.9 Å) < OAc^−^ (7.4 Å), followed by the difference between the two cations, K^+^ (6.6 Å) < Na^+^ (7.2 Å); see Figure 3.

**Figure 6:**
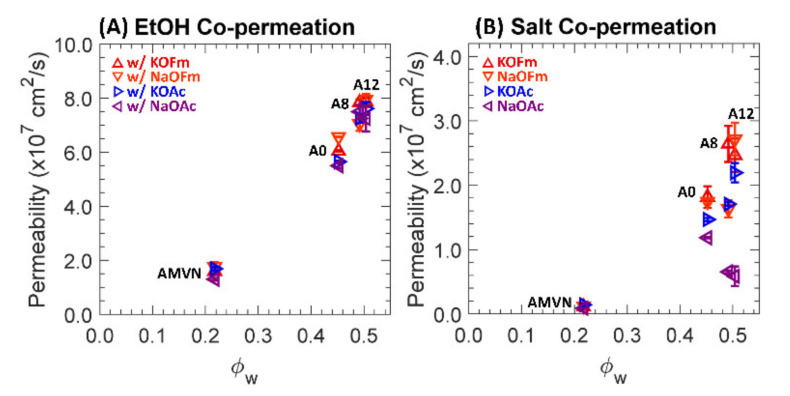
(**A**) Permeabilities to EtOH in co-permeation with KOFm (△, red), NaOFm (▽, orange), KOAc (▷, blue) and NaOAc (◁, purple). (**B**) Permeabilities to KOFm (△, red), NaOFm (▽, orange), KOAc (▷, blue) and NaOAc (◁, purple) in co-permeation with EtOH. Each data point is the average of 3 experiments, with error bars corresponding to the standard deviation.

Two-component permeabilities to EtOH and carboxylate salts (KOFm, NaOFm, KOAc and NaOAc) of a charge-neutral A0 and positively charged A8, A12 and AMVN films in HCO_3_ form are shown in Figure 6, where (A) and (B) are scaled differently; see Supporting Information Table S2 for values.

**Figure 7:**
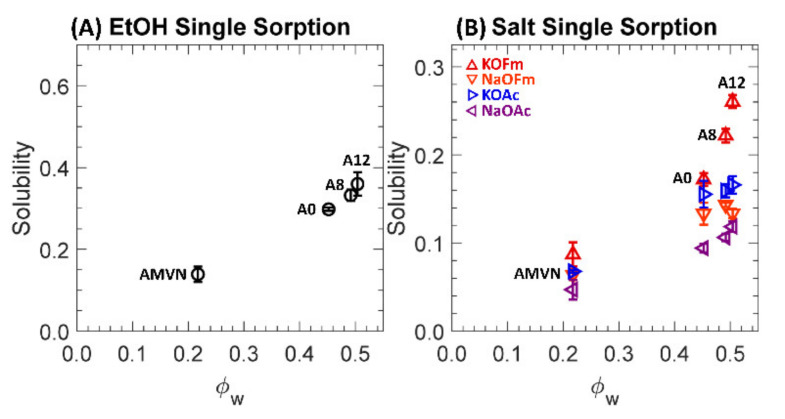
(**A**) Solubilities to EtOH, ○, in single sorption. (**B**) Solubilities to KOFm (△, red), NaOFm (▽, orange), KOAc (▷, blue) and NaOAc (◁, purple) in single sorption. Each data point is the average of 3 experiments, with error bars corresponding to the standard deviation.

**Figure 8:**
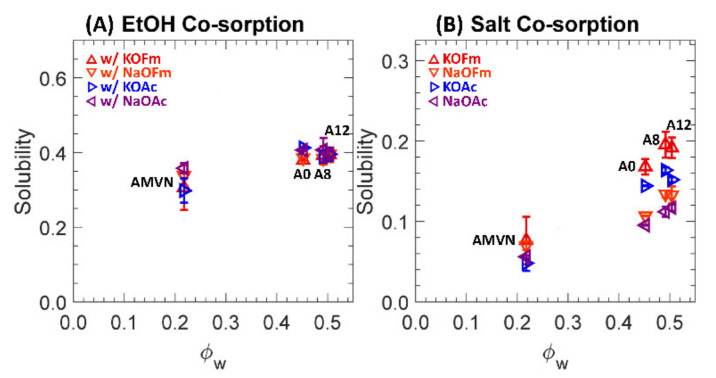
(**A**) Solubilities to EtOH in co-sorption with KOFm (△, red), NaOFm (▽, orange), KOAc (▷, blue) and NaOAc (◁, purple). (**B**) Solubilities to KOFm (△, red), NaOFm (▽, orange), KOAc (▷, blue) and NaOAc (◁, purple) in co-sorption with EtOH. Each data point is the average of 3 experiments, with error bars corresponding to the standard deviation.

In co-permeation, the permeabilities of AMVN to EtOH are increased by 1.7 times, while those of PEGDA-based films are essentially the same. This is largely due to the differences in sorption, which are described below; see Figure 7 and Figure 8. Interestingly, QA^+^-free A0 permeabilities to NaOAc and KOFm are decreased by 1.1 times, on average, in co-permeation, while those to NaOFm and KOAc are increased by 1.2 and 1.1 times, respectively, on average. However, QA^+^-containing A8 and A12 permeabilities to NaOAc, KOFm, NaOFm and KOAc all decrease, by 2.2, 1.4, 1.3 and 1.1 times, respectively, on average. To rationalize this behavior, we conjecture the permeation of carboxylate salts to be dependent on the polyatomic carboxylate anions over the cations. Consequently, electrostatic attraction (i.e., counterion condensation [18]) between the bound quaternary ammonium (QA^+^) and mobile carboxylate anions (OFm^−^ and OAc^−^) can be suppressed by co-permeation with EtOH (i.e., charge screening [29,30,32]); see Figure 1. As a result, the overall salt permeabilities of QA^+^-containing A8 and A12 are decreased in co-permeation with EtOH. Similarly, AMVN permeabilities to OFm^−^-containing salts (KOFm and NaOFm) are decreased by three times, while those to OAc^−^-containing salts (KOAc and NaOAc) are similar. More details of this behavior will be discussed below in the section on diffusion.

### 3.4. Sorption

One-component solubilities to EtOH and carboxylate salts (KOFm, KOAc, NaOFm and NaOAc) of a charge-neutral A0 and positively charged A8, A12 and AMVN films in HCO_3_^−^ form are shown in Supporting Information Table S3 and Figure 7, where (A) and (B) are scaled differently. Generally, the volumes of AMVN films after sorption in all external solutions were slightly increased (6–9%), and the volumes of PEGDA-based films were essentially the same (within 3%) or slightly increased (up to 9%) after sorption; see Supporting Information Table S7.

Generally, EtOH solubilities are higher than those of salt solubilities by 1.7 times, on average, indicating that EtOH uptake is preferred in these films (*ϕ_w_*: 0.2–0.5) over the uptake of carboxylate salts. We observed similar behavior in a previous investigation of cation exchange membranes (CEM) [29,32,50], where the alcohol (MeOH and EtOH) solubilities were higher than the carboxylate (NaOFm and NaOAc) solubilities. However, contrary to our previous investigations of CEMs, the EtOH concentrations in the AEMs here (A0, A8, A12 and AMVN) after sorption in the external solution (1 M EtOH) were less than those of the external solution, such that EtOH is less preferred in these films over the external solution; see Supporting Information Table S8. 

For PEGDA-based films (A0, A8 and A12), the carboxylate salt solubilities were in the order of KOFm > KOAc > NaOFm > NaOAc. This result indicates that the salts with K^+^ (KOFm and KOAc) are preferred over the salts with Na^+^ (NaOFm and NaOAc). A similar result was observed by Jang et al. [41], where the potassium chloride (KCl) solubilities of crosslinked PEGDA films were higher than their sodium chloride (NaCl) solubilities. They proposed that it is easier for K^+^ ions to bind with PEG as they can directly interact with the dipole moment of the ether oxygen group in the absence of a strongly bound hydration layer due to the relatively low surface charge density (0.072 mC/cm^2^), while it is more difficult for Na^+^ ions to interact with the dipole moment with a strongly bound hydration layer due to the relatively high surface charge density (0.142 mC/cm^2^) [41]. Similarly, Sartori et al. reported the binding constant of K^+^ to ethylene oxide to be higher than that of Na^+^ to ethylene oxide [69]. This result also suggests that the salts with OFm^−^ (KOFm and NaOFm) are preferred over the salts with OAc^−^ (KOAc and NaOAc). A possible cause of this difference between the solubilities of OFm^−^ and OAc^−^ is the effect of molecular size, where the larger OAc^−^ (7.4 Å) would experience more steric hindrance from the polymer structure than smaller OFm^−^ (5.9 Å) (Figure 3) [32,41,51,70]. For AMVN, the salt solubilities are in the order of KOFm > NaOFm > KOAc > NaOAc; the order between NaOFm and KOAc is changed. This indicates that the effect of carboxylates (OFm^−^ > OAc^−^) is more apparent than the effect of cations (K^+^ > Na^+^). This is likely due to the fact that the polystyrene-divinylbenzene (PS-DVB) backbone in AMVN does not contain functional groups with a strong dipole moment as ether oxygen groups in PEG; see Figure 2.

Two-component solubilities to EtOH and carboxylate salts (KOFm, NaOFm, KOAc and NaOAc) of a charge-neutral A0 and positively charged A8, A12 and AMVN films in HCO_3_ form are shown in Figure 8, where (A) and (B) are scaled differently.

In co-sorption, PEGDA-based films’ (A0, A8 and A12) solubilities to EtOH are increased by 1.3, 1.2 and 1.1 times, on average, respectively, while those to salts are decreased by 1.1 times, on average. Again, EtOH is preferentially sorbed into PEGDA-based films over carboxylate salts in co-sorption. Our group reported similar behavior [32] for a series of crosslinked PEGDA (same as A0) and sulfonate-bearing PEGDA-based CEMs (similar to A8 and A12, but a CEM with negatively charged sulfonate groups). For those CEMs, solubilities to alcohols in co-sorption with a carboxylate salt were increased by 1.2 and 1.1 times, on average, respectively, while those to salts in co-sorption were essentially the same. A possible cause of this behavior is the difference in hydrophobicity [71]. While both EtOH and carboxylate salts are hydrophilic, as they bear an alcohol group (-OH) and charged groups (i.e., a carboxylate^−^ and either K^+^ or Na^+^), respectively, the carboxylate salts are relatively more hydrophilic due to the hydration of the charge groups and, therefore, their interaction might be less preferred with a polymer structure (relatively hydrophobic).

In co-sorption, AMVN solubilities to EtOH are increased by 2.1 times, on average, while those to salts are essentially the same. This is contrary to behavior reported for CEMs [32], where the solubilities of a commercial perfluorosulfonic acid (PFSA) CEM, Nafion^®^ 117, to alcohols in co-sorption with a carboxylate salt were decreased by 1.1 times, on average. As current state-of-the-art IEMs, AMVN and Nafion^®^ 117 share some common characteristics, such as a hydrophobic backbone (i.e., PS-DVB and PF), similar IEC (i.e., 1.5 and 0.9 meq/g) and similar water volume fraction (i.e., 0.22 and 0.25 [30]). To rationalize this difference in transport behavior between AEM and CEM, we conjecture a potential repulsive interaction between bound QA^+^ and mobile EtOH in single sorption (i.e., AEM direct ethanol fuel cells, DEFC [3]), which might be interfered with by mobile carboxylate anions as they are attracted to the bound QA^+^ and screen the interaction between the QA^+^ and EtOH. Nevertheless, the increase in EtOH sorption in AEM is a concerning behavior for CO_2_ reduction cells [8,9,10,12,16] and, therefore, it would be appropriate to make efforts to suppress this behavior in the design of AEMs for CO_2_ reduction [7,28].

### 3.5. Diffusion

**Figure 9:**
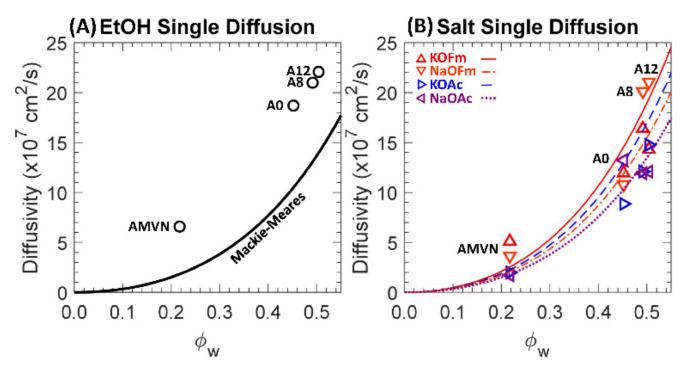
(**A**) Diffusivities to EtOH, ○, in single diffusion. The solid line is the Mackie–Meares’ fit. (**B**) Diffusivities to KOFm (△, red), NaOFm (▽, orange), KOAc (▷, blue) and NaOAc (◁, purple) in single diffusion. The lines are the Mackie–Meares’ fits, KOFm (solid line, red), NaOFm (dot-dashed, orange), KOAc (dashed, blue) and NaOAc (dotted, purple).

One-component diffusivities to EtOH and carboxylate salts (KOFm, KOAc, NaOFm and NaOAc) of a charge-neutral A0 and positively charged A8, A12 and AMVN films in HCO_3_ form were calculated using the solution-diffusion relationship (Equation (1)) and the results are shown in Figure 9; see Supporting Information Table S4 for values. The Mackie–Meares model was used to correlate the diffusivities with the water volume fraction of each membrane (Equation (8)).

The calculated EtOH diffusivities are higher than those estimated by the Mackie–Meares model by 2.1 times, on average, showing a similar result to MeOH diffusivities reported elsewhere [42]. This under-prediction of alcohol diffusivity has been observed previously and attributed to the Mackie–Meares model being initially devised for ionic species [60]. The relative difference in the calculated and the estimated diffusivities was larger in AMVN. For instance, the calculated EtOH diffusivity of AMVN was 3.6 times higher than the estimated value from the Mackie–Meares model, while those of PEGDA-based films were 1.7 times higher. A contribution to the significantly low estimation for AMVN is made by the inherent weakness of the model at low water volume fraction. For instance, the model implies that the solutes become immobile at zero water volume fraction, which is not true as they can diffuse through the backbone structure [18,60]. Another contribution is the increase in the volume fraction of solution, *ϕ_s_* [42]:

(11) $$\phi_{s}=\frac{V_{s}-\left(W_{d}/\rho_{L}\right)}{V_{s}}$$

where $W_{d}$ is the mass of the dried film, *ρ_L_* is the density of water, and vs. is the volume of the swollen film after sorption. To determine *V_s_*, the film surface area was extracted from digital photographs and the thickness was measured using a digital caliper; see Supporting Information Table S9 for values. As the solution volume fraction was higher than the water volume fraction by 1.1 times, the AMVN diffusivities can be closer to the Mackie–Meares’ fit as this would constitute a rightward shift in the values in Figure 8A.

The salt diffusivities are closer to the Mackie–Meares’ fits; see Figure 8B. To calculate the diffusivities of salts in water (*D_0,i_*, Equation (8)), we assume the diffusivities of a salt consisting of monovalent ions (i.e., Na^+^ and Cl^−^ for NaCl) and that the salt diffusivity is close to the average diffusivities of the two ions (mobility-weighted average diffusivity [72]). For instance, using the reported diffusivities of Na^+^, K^+^, Cs^+^ and Cl^−^ in water (1.33 × 10^−5^, 1.96 × 10^−5^, 2.06 × 10^−5^ and 2.03 × 10^−5^ cm^2^/s, respectively [63]), the estimated diffusivities of NaCl, KCl and CsCl in water are 1.68 × 10^−5^, 2.00 × 10^−5^ and 2.04 × 10^−5^ cm^2^/s, respectively, and these are close to the reported diffusivities for these salts: 1.61 × 10^−5^ [73], 1.99 × 10^−5^ and 2.04 × 10^−5^ cm^2^/s, respectively. Here, using the reported diffusivities of K^+^, Na^+^, OFm^−^ and OAc^−^ in water, 1.96 × 10^−5^, 1.33 × 10^−5^, 1.45 × 10^−5^ and 1.09 × 10^−5^ cm^2^/s [63,64], repectively, we estimate the diffusivites of KOFm, KOAc, NaOFm and NaOAc in water as 1.71 × 10^−5^, 1.52 × 10^−5^, 1.39 × 10^−5^ and 1.21 × 10^−5^ cm^2^/s, respectively.

Generally, salt diffusivities to AMVN are higher than the estimated diffusivities by 1.4 times, on average, while those to PEGDA-based films are essentially the same. Again, a contribution to the low estimation for AMVN is presumably made by the inherent weakness of the model at low water volume fraction. For AMVN, the calculated diffusivities to K^+^-containing salts (KOFm and KOAc) are higher than the estimated diffusivities by 1.5 times, on average, while those to Na^+^-containing salts (NaOFm and NaOAc) are higher than the estimated diffusivities by 1.4 times, on average. The calculated diffusivities of PEGDA-based films to Na^+^-containing salts are higher than the estimated diffusivities by 1.1 times, on average, while those to K^+^-containing salts are lower than the estimated diffusivities by 1.3 times, on average.

**Figure 10:**
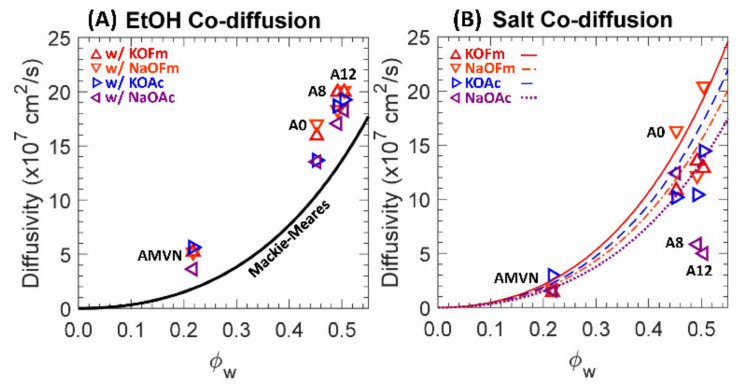
(**A**) Diffusivities to EtOH, ○, in co-diffusion with KOFm (△, red), NaOFm (▽, orange), KOAc (▷, blue) and NaOAc (◁, purple). The solid line is the Mackie–Meares’ fit. (**B**) Diffusivities to KOFm (△, red), NaOFm (▽, orange), KOAc (▷, blue) and NaOAc (◁, purple) in co-diffusion with EtOH. The lines are the Mackie–Meares’ fits, KOFm (solid line, red), NaOFm (dot-dashed, orange), KOAc (dashed, blue) and NaOAc (dotted, purple).

Two-component diffusivities to EtOH and carboxylate salts (KOFm, NaOFm, KOAc and NaOAc) of a charge-neutral A0 and positively charged A8, A12 and AMVN films in HCO_3_^−^ form are shown in Figure 10 along with predicted diffusivities using the Mackie–Meares model.

In co-diffusion, the EtOH diffusivities are decreased by 1.2 times, on average. Our group reported a similar result for CEMs (PEGDA-AMPS and Nafion^®^ 117) [32], where both MeOH and EtOH diffusivities were decreased in co-diffusion with a carboxylate salt (either NaOFm or NaOAc). To rationalize this behavior, we conjectured there to be competitive diffusion between the co-solutes [32], and that the diffusional path of a fast-diffusing diffusant can be interfered with by a slow-diffusing co-diffusant. A similar diffusant–diffusant interaction is likely of consequence for the EtOH diffusion with these carboxylate salts in the AEMs studied here. For PEGDA-based films (A0, A8 and A12), the decrease in EtOH diffusivities was more apparent in co-diffusion with OAc^−^-containing salts (KOAc and NaOAc). While EtOH diffusivities in co-diffusion with OFm^−^-containing salts (KOFm and NaOFm) were decreased by 1.11 times, on average, those with OAc^−^-containing salts were decreased by 1.23 times, on average. The impact of the difference in carboxylate anion (as stated above) was more apparent than the impact from the difference in cation, where EtOH diffusivities in co-diffusion with K^+^-containing salts (KOFm and KOAc) and Na^+^-containing salts (NaOFm and NaOAc) were decreased by 1.15 and 1.18 times, on average, respectively. A possible cause is the hydrated diameter of OFm^−^ (5.9 Å) being significantly less than that of OAc^−^ (7.4 Å) and, therefore, the larger-diameter anion correlates to a larger impediment to the fast-diffusing EtOH, while the differences in the hydrated diameters of K^+^ (6.6 Å) and Na^+^ (7.2 Å) are relatively small (Figure 3). For AMVN, the impact of the difference in cation was more apparent than the impact of the difference in anion. While EtOH diffusivities in co-diffusion with K^+^-containing salts were decreased by 1.39 times, on average, those with Na^+^-containing salts were increased by 1.07 times, on average. EtOH diffusivities in co-diffusion with OFm^−^-containing salts and OAc^−^-containing salts were decreased by 1.13 and 1.10 times, on average, respectively. To rationalize the increase in EtOH diffusivity in co-diffusion with Na^+^-containing salts, we conjecture that the diffusional path of EtOH is less interfered with by the salts with Na^+^. As the surface charge density of Na^+^ is higher than that of K^+^, the electrostatic repulsion from bound QA^+^ to Na^+^ can be higher than that to K^+^. Consequently, more salts with Na^+^ might diffuse away from the bound water region and, therefore, more EtOH can diffuse near the bound water region, which will have a lesser effect on the fast-diffusing EtOH. On the other hand, the diffusional path of EtOH and the salts with K^+^ might be overlapping and, therefore, more salts can impede the EtOH diffusion.

In co-diffusion with EtOH, QA^+^-free A0 (crosslinked PEGDA) diffusivities to carboxylate salts were slightly increased by 1.1 times, on average, and a similar result was reported elsewhere [32]. This behavior is partially due to flux coupling [12,38] between fast-diffusing EtOH and slow-diffusing carboxylate salts. The diffusivities of QA^+^-containing AEMs (A8, A12 and AMVN) to carboxylate salts were decreased by 1.3 times, on average, in co-diffusion with EtOH. We observed the opposite behavior in sulfonate (SO_3_^−^)-containing CEMs [32], where the diffusivities of SO_3_^−^-containing PEGDA-AMPS (equivalent to A8 and A12, but with SO_3_^−^ group) and Nafion^®^ 117 were increased by 1.4 times, on average. To rationalize this behavior, we conjectured a partial charge screening [27,31] by a co-diffusing alcohol [29,30] such that the electrostatic repulsion (Donnan exclusion; [36]) between bound SO_3_^−^ and mobile carboxylate anions (co-ions in CEM) is diminished and, therefore, the overall salt diffusivity is increased. Typically, the salt diffusion in an IEM (while maintaining the charge neutrality) is often limited by the co-ion (electrostatic repulsion) [18] and, therefore, the charge screening between the bound charge and the co-ion was in line with the traditional understanding of salt diffusion in IEM. If the co-ion (either K^+^ or Na^+^) of these carboxylate salts shows a significant impact on the diffusion through AEMs, then the charge screening by alcohol might be assisting the overall salt diffusion (rather than suppressing, as seen in this investigation). This leads to a conjecture that the impact of K^+^ or Na^+^ is not apparent and a possible contribution is the difference in the kinetic diameters of cations and carboxylate anions. As polyatomic anions, the kinetic diameters of carboxylate anions are significantly larger than those of K^+^ and Na^+^. These differences may impact the hydration shells of the cations (unlike those with a smaller monovalent anion, Cl^−^; [44]). Taken together, the electrostatic attraction (counterion condensation theory; [18,20,37]) between bound QA^+^ and mobile carboxylate anions (counterions in AEM) can be dominant over cations and can be diminished through a partial charge screening by the co-diffusing EtOH and, therefore, the overall salt diffusivity is decreased as the diffusivity of the condensed counterion [18] is diminished; see Figure 1. Overall, these changes in interactions suggest that differences in diffusion behavior from the above-described interactions are a primary driver of changes in membrane diffusivities to carboxylate salts in single and co-diffusion with alcohols and perhaps for understanding the co-diffusion of other complex mixtures through IEMs.

## 4. Conclusions

A QA^+^-free PEGDA (A0), two QA^+^-containing PEGDA-APTA (A8 and A12) and Selemion^®^ AMVN (AMVN) were investigated for their transport and co-transport behavior when challenged with carboxylate ions, EtOH and mixtures of carboxylate ions and EtOH. Permeabilities and solubilities to EtOH or carboxylate salt (either KOFm, KOAc, NaOFm or NaOAc) were measured both by themselves and in co-transport. Solute diffusivities for each case were then calculated using the solution-diffusion model, where, generally, EtOH exhibited higher solubility and diffusivity than the carboxylate salts in all films. A charge screening behavior is conjectured based on the assumption that the diffusion of a carboxylate salt is dependent on the polyatomic carboxylate anion over the cation. The carboxylate salt diffusivities of AEMs (A8, A12 and AMVN) are decreased in co-diffusion with EtOH, which we ascribe to the screening of the electrostatic attraction by the co-diffusing EtOH (charge screening). Overall, multi-component transport in ion exchange membranes is a highly complex system as various mobile components (i.e., cation, uncondensed carboxylate anions, condensed carboxylate anions, EtOH and bulk water) are permeating in various fixed components (i.e., QA^+^, polymer backbone and bound water) and the array of interactions between solutes and between solutes and the membrane are dynamic and complicated. Therefore, while this investigation extends our understanding of the transport and co-transport behavior of select solutes (carboxylate ions and EtOH), more fundamental investigations are needed to further develop our understanding of the transport behavior of complex mixtures in polymer membranes.

## Figures and Tables

**Table 1:** Membrane properties of pre-polymerization mixtures.

	APTA ^1^ (mol%)	PEGDA (g)	APTA (g)	Water (g)	HCPK (g)
A0	0	8.00	0.00	2.00	0.008
A8	8	7.80	0.20	2.00	0.008
A12	12	7.69	0.31	2.00	0.008

^1^ APTA = mol of APTA/(mol of PEGDA + mol of APTA) × 100%.

**Table 2:** Water uptake, dry polymer density and water volume fraction of all films.

	**Water Uptake,** $\omega_{w}$ **(Water g/Dry Polymer g·100%)**		**Dry Polymer** **Density**, $\rho_{p}$**(g/mL)**		**Water Volume** **Fraction, *ϕ_w_***	
	Cl^−^	HCO_3_^−^	Cl^−^	HCO_3_^−^	Cl^−^	HCO_3_^−^
A0	68 ± 1		1.22 ± 0.01		0.45	
A8	70 ± 0	77 ± 0	1.24 ± 0.02	1.25 ± 0.05	0.46	0.49
A12	72 ± 2	83 ± 1	1.21 ± 0.00	1.22 ± 0.01	0.46	0.50
AMVN	18 ± 1	27 ± 0	1.01 ± 0.00	1.02 ± 0.01	0.15	0.22

**Table 3:** Ionic conductivity and IEC of all films.

	Ionic Conductivity (σ, mS/cm)		IEC (meq/g Dry Polymer)	
	Cl^−^	HCO_3_^−^	Cl^−^	HCO_3_^−^
A8	1.0 ± 0.0	0.9 ± 0.0	0.121	0.125 ± 0.004
A12	1.2 ± 0.0	0.8 ± 0.0	0.187	0.190 ± 0.001
AMVN	7.0 ± 0.0	4.1 ± 0.0	1.5 ^1^	-

^1^ Reported by the manufacturer.

## Data Availability

Data not contained within this manuscript and Supporting Information is available upon direct request to the corresponding author.

## Supplementary Materials

The following are available online at https://www.mdpi.com/article/10.3390/polym13172885/s1, Figure S1. Exemplary (A) Photopolymerization of a pre-polymerization mixture. (B) A hydrated crosslinked film. (C) Selemion^®^ AMVN. Table S1. Solute diffusivities of select species in water (×10^5^ cm^2^/s) at 25 °C in the dilute condition. Table S2. Diffusive permeabilities (×10^7^ cm^2^/s) of A0, A8, A12 and AMVN to EtOH and carboxylate salts in single and EtOH-carboxylate mixture. Table S3. Solubilities of A0, A8, A12 and AMVN to EtOH and carboxylate salts in single and EtOH-carboxylate mixture. Table S4. Diffusivities (×10^7^ cm^2^/s) of A0, A8, A12 and AMVN to EtOH and carboxylate salts in single and EtOH-carboxylate mixture. Table S5. Weight percent (wt.%) of AEMs (A8, A12 and AMVN) in Cl^−^ and HCO_3_^−^ form. Figure S2. Exemplary EDS spectra for AEMs, (A,B) A8, (C,D) A12, (E,F) AMVN, in (A,C,E) Cl^−^ and (B,D,F) HCO_3_^−^ form. Table S6. Normalized film thickness to hydrated membrane after permeability measurements. Table S7. Volume of hydrated films and volume of swollen films (mm^3^) after sorption experiments measured from photographs and a digital caliper. Normalized to the volume of the hydrated films. Table S8. Volume fraction among the solution, EtOH (ϕ_e_)-carboxylate salt (ϕ_c_), inside the membranes after sorption experiments, where the remaining is the volume fraction of water (ϕ_w_) from the solution. Table S9. Water volume fractions (ϕ_w_) and solution volume fractions (ϕ_s_) of films after sorption experiments, where the remaining is the polymer volume fraction (ϕ_p_) from the dry polymer density.
