# Supplementary material for: Transport and Co-Transport of Carboxylate Ions and Ethanol in Anion Exchange Membranes
Source: Polymers (Basel). 2021 Aug 27;13(17):2885. doi: 10.3390/polym13172885 (PMC8433790; doi:10.3390/polym13172885)
Supplement: Supplementary file 1 [file polymers-13-02885-s001.zip › polymers-1332527-supplementary.pdf]

Supporting information for:

Transport and co-transport of carboxylate ions and ethanol in anion exchange membranes

Jung Min Kim[1], Yi-hung Lin[1], Brock Hunter[1], Bryan S. Beckingham[1,*]

[1]Department of Chemical Engineering, Auburn University, Auburn, AL 36849, United States

*Corresponding Author: Tel: +1 (334) 844-2036

E-mail Address: bsb0025@auburn.edu (Bryan S. Beckingham)

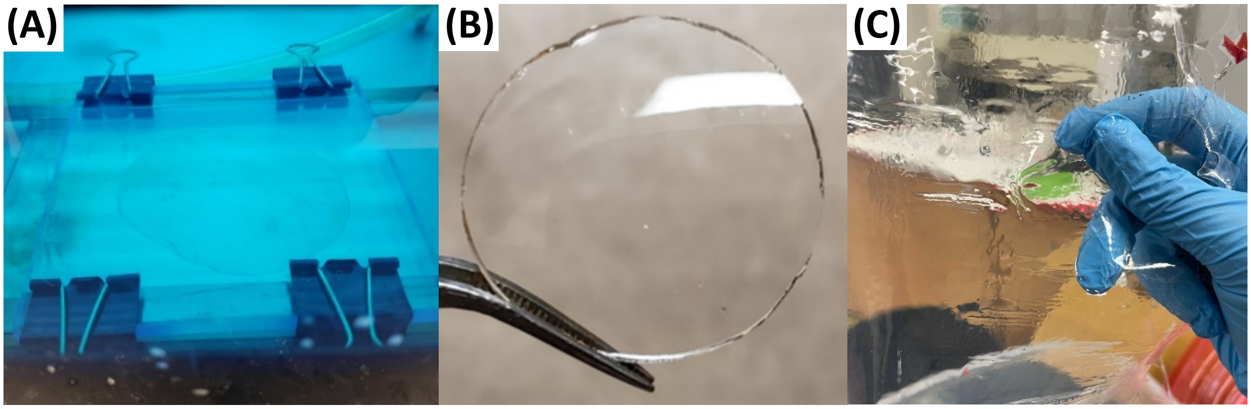

Figure S1. (A) Photopolymerization of a prepolymerization mixture. (B) A hydrated crosslinked film. (C) Selemion® AMVN.

Table S1. Solute diffusivities of select species in water (× 10^5 cm^2/s) at 25 ºC in the dilute condition.

Solute	Diffusivity in water
Water	2.45 [1]
EtOH	1.23 [2]
OFm^-	1.454 [3]
OAc^-	1.089 [3,4]
K^+	1.957 [4]
Na^+	1.334 [4]

Table S2. Diffusive permeabilities (× 10^7 cm^2/s) of A0, A8, A12, and AMVN to EtOH and carboxylate salts in single and EtOH-carboxylate mixture.

A0		K	Na
Single	OFm	2.06 ± 0.11	1.44 ± 0.14
EtOH	OFm	1.82 ± 0.17	1.75 ± 0.03
Single	OAc	1.38 ± 0.03	1.25 ± 0.01
EtOH	OAc	1.47 ± 0.02	1.18 ± 0.01

A0	EtOH
Single	5.57 ± 0.27
KOFm	6.04 ± 0.03
NaOFm	6.56 ± 0.01
KOAc	5.65 ± 0.04
NaOAc	5.50 ± 0.08

A8		K	Na
Single	OFm	3.64 ± 0.36	2.89 ± 0.16
EtOH	OFm	2.64 ± 0.28	1.63 ± 0.13
Single	OAc	1.93 ± 0.07	1.27 ± 0.04
EtOH	OAc	1.71 ± 0.03	0.65 ± 0.02

A8	EtOH
Single	6.97 ± 0.34
KOFm	7.84 ± 0.12
NaOFm	7.05 ± 0.26
KOAc	7.25 ± 0.17
NaOAc	7.50 ± 0.25

A12		K	Na
Single	OFm	3.73 ± 0.24	2.81 ± 0.16
EtOH	OFm	2.46 ± 0.26	2.72 ± 0.25
Single	OAc	2.45 ± 0.13	1.43 ± 0.02
EtOH	OAc	2.19 ± 0.15	0.58 ± 0.15

A12	EtOH
Single	7.94 ± 0.02
KOFm	7.87 ± 0.19
NaOFm	7.95 ± 0.21
KOAc	7.62 ± 0.23
NaOAc	7.25 ± 0.48

AMVN		K	Na
Single	OFm	0.44 ± 0.02	0.23 ± 0.03
EtOH	OFm	0.11 ± 0.01	0.12 ± 0.01
Single	OAc	0.13 ± 0.02	0.08 ± 0.00
EtOH	OAc	0.14 ± 0.02	0.09 ± 0.00

AMVN	EtOH
Single	0.92 ± 0.05
KOFm	1.60 ± 0.14
NaOFm	1.75 ± 0.17
KOAc	1.68 ± 0.06
NaOAc	1.30 ± 0.05

Table S3. Solubilities of A0, A8, A12, and AMVN to EtOH and carboxylate salts in single and EtOH-carboxylate mixture.

A0		K	Na
Single	OFm	0.172 ± 0.007	0.133 ± 0.013
EtOH	OFm	0.168 ± 0.010	0.107 ± 0.000
Single	OAc	0.155 ± 0.015	0.094 ± 0.005
EtOH	OAc	0.144 ± 0.001	0.095 ± 0.000

A0	EtOH
Single	0.298 ± 0.004
KOFm	0.379 ± 0.001
NaOFm	0.386 ± 0.001
KOAc	0.413 ± 0.001
NaOAc	0.407 ± 0.001

A8		K	Na
Single	OFm	0.222 ± 0.008	0.143 ± 0.002
EtOH	OFm	0.195 ± 0.016	0.134 ± 0.004
Single	OAc	0.159 ± 0.007	0.106 ± 0.004
EtOH	OAc	0.164 ± 0.004	0.112 ± 0.006

A8	EtOH
Single	0.332 ± 0.014
KOFm	0.393 ± 0.025
NaOFm	0.385 ± 0.016
KOAc	0.388 ± 0.016
NaOAc	0.407 ± 0.032

A12		K	Na
Single	OFm	0.260 ± 0.007	0.134 ± 0.006
EtOH	OFm	0.192 ± 0.013	0.133 ± 0.010
Single	OAc	0.166 ± 0.010	0.119 ± 0.006
EtOH	OAc	0.152 ± 0.003	0.117 ± 0.004

A12	EtOH
Single	0.360 ± 0.029
KOFm	0.394 ± 0.020
NaOFm	0.395 ± 0.002
KOAc	0.396 ± 0.017
NaOAc	0.396 ± 0.014

AMVN		K	Na
Single	OFm	0.087 ± 0.014	0.064 ± 0.006
EtOH	OFm	0.076 ± 0.030	0.070 ± 0.006
Single	OAc	0.068 ± 0.002	0.047 ± 0.011
EtOH	OAc	0.048 ± 0.010	0.056 ± 0.002

AMVN	EtOH
Single	0.139 ± 0.019
KOFm	0.306 ± 0.060
NaOFm	0.274 ± 0.007
KOAc	0.395 ± 0.005
NaOAc	0.168 ± 0.004

Table S4. Diffusivities (× 10^7 cm^2/s) of A0, A8, A12, and AMVN to EtOH and carboxylate salts in single and EtOH-carboxylate mixture.

A0		K	Na
Single	OFm	11.9	10.8
EtOH	OFm	10.8	16.3
Single	OAc	8.9	13.2
EtOH	OAc	10.2	12.4

A0	EtOH
Single	18.7
KOFm	15.9
NaOFm	17.0
KOAc	13.7
NaOAc	13.5

A8		K	Na
Single	OFm	16.4	20.1
EtOH	OFm	13.5	12.2
Single	OAc	12.1	11.9
EtOH	OAc	10.4	5.8

A8	EtOH
Single	21.0
KOFm	20.0
NaOFm	18.3
KOAc	18.7
NaOAc	17.1

A12		K	Na
Single	OFm	14.3	21.1
EtOH	OFm	12.9	20.4
Single	OAc	14.8	12.1
EtOH	OAc	14.4	5.0

A12	EtOH
Single	22.1
KOFm	20.0
NaOFm	20.1
KOAc	19.3
NaOAc	18.3

AMVN		K	Na
Single	OFm	5.1	3.6
EtOH	OFm	1.4	1.7
Single	OAc	2.0	1.7
EtOH	OAc	3.0	1.6

AMVN	EtOH
Single	6.6
KOFm	5.2
NaOFm	6.4
KOAc	4.3
NaOAc	7.7

Table S5. Weight percent (wt.%) of AEMs (A8, A12, and AMVN) in Cl^- and HCO_3^- forms.

		Measured			Theoretical*		
		C	O	Cl	C	O	Cl
Cl-form	A8	57.8 ± 0.7	41.6 ± 0.6	0.6 ± 0.1	59.9	37.8	2.3
	A12	57.7 ± 0.3	41.1 ± 0.3	1.2 ± 0.1	59.8	36.8	3.4
	AMVN	89.6 ± 0.4	6.2 ± 0.3	4.1 ± 0.1	-	-	-
HCO_3- form	A8	59.5 ± 0.2	40.5 ± 0.2	-	59.7	40.3	-
	A12	58.9 ± 0.5	41.1 ± 0.5	-	59.6	40.4	-
	AMVN	91.3 ± 0.3	8.7 ± 0.3	-	-	-	-

*Theoretical values of A8 and A12 were calculated based on the compositions in prepolymerization mixtures.

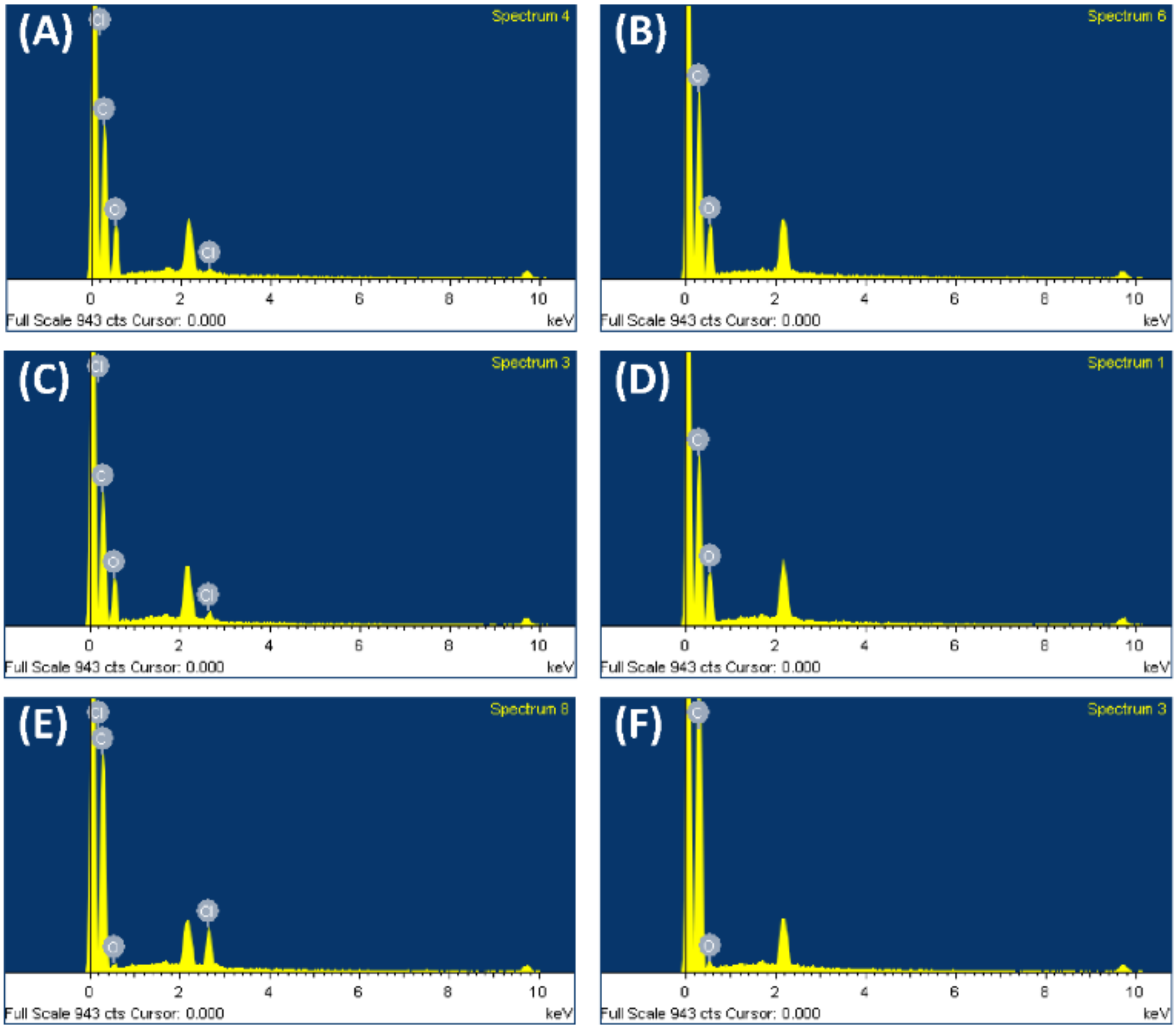

Figure S2. Exemplary EDS spectra for AEMs, (A,B) A8, (C,D) A12, (E,F) AMVN, in (A,C,E) Cl^- and (B,D,F) HCO_3^- forms.

Table S6. Normalized film thickness to hydrated membrane after permeability measurements.

	AMVN	A0	A8	A12
Hydrated	1.00	1.00	1.00	1.00
1. EtOH	1.05	0.93	0.91	0.85
2. KOFm	0.99	0.93	0.90	0.83
3. NaOFm	0.95	0.92	0.88	0.85
4. KOAc	1.02	0.91	0.90	0.83
5. NaOAc	1.05	0.93	0.89	0.83
6. EtOH/KOFm	1.01	0.92	0.91	0.87
7. EtOH/NaOFm	1.03	0.93	0.90	0.85
8. EtOH/KOAc	1.03	0.93	0.90	0.84
9. EtOH/NaOAc	1.03	0.93	0.90	0.84

Table S7. Volume of hydrated films and volume of swollen films (mm^3) after sorption experiments measured from photographs and a digital caliper. Normalized to the volume of the hydrated films.

	A0	A8	A12	AMVN
Hydrated	1.000	1.000	1.000	1.000
1. EtOH	0.988	0.904	0.899	1.085
2. KOFm	0.976	0.926	0.926	1.081
3. NaOFm	0.994	0.885	0.959	1.078
4. KOAc	0.968	0.885	0.880	1.068
5. NaOAc	0.946	0.899	0.876	1.084
6. EtOH/KOFm	1.090	1.016	0.993	1.070
7. EtOH/NaOFm	1.074	0.975	0.975	1.082
8. EtOH/KOAc	1.022	0.980	1.005	1.093
9. EtOH/NaOAc	1.002	0.969	0.984	1.078

Table S8. Volume fraction among the solution, EtOH (ϕ_e)-carboxylate salt (ϕ_c), inside the membranes after sorption experiments, where the remaining is the volume fraction of water (ϕ_w) from the solution.

	External, 1 M		AMVN		A0		A8		A12	
	ϕ_e	ϕ_c	ϕ_e	ϕ_c	ϕ_e	ϕ_c	ϕ_e	ϕ_c	ϕ_e	ϕ_c
1. EtOH	0.058	-	0.038	-	0.033	-	0.051	-	0.047	-
2. KOFm	-	0.044	-	0.017	-	0.016	-	0.025	-	0.025
3. NaOFm	-	0.035	-	0.010	-	0.009	-	0.014	-	0.010
4. KOAc	-	0.063	-	0.022	-	0.018	-	0.027	-	0.024
5. NaOAc	-	0.054	-	0.012	-	0.010	-	0.015	-	0.015
6. EtOH/KOFm	0.058	0.044	0.044	0.015	0.076	0.014	0.051	0.019	0.046	0.017
7. EtOH/NaOFm	0.058	0.035	0.045	0.008	0.081	0.010	0.053	0.011	0.047	0.010
8. EtOH/KOAc	0.058	0.063	0.051	0.019	0.070	0.012	0.053	0.024	0.046	0.019
9. EtOH/NaOAc	0.058	0.054	0.052	0.011	0.087	0.013	0.056	0.014	0.047	0.013

Table S9. Water volume fractions (ϕ_w) and solution volume fractions (ϕ_s) of films after sorption experiments, where the remaining is the polymer volume fraction (ϕ_p) from the dry polymer density.

	AMVN, ϕ_s	A0, ϕ_s	A8, ϕ_s	A12, ϕ_s
Water volume fractions, ϕ_w	0.217	0.452	0.492	0.504
1. EtOH	0.245	0.452	0.382	0.446
2. KOFm	0.242	0.445	0.397	0.462
3. NaOFm	0.240	0.455	0.369	0.481
4. KOAc	0.233	0.441	0.369	0.434
5. NaOAc	0.244	0.428	0.380	0.432
6. EtOH/KOFm	0.235	0.503	0.451	0.499
7. EtOH/NaOFm	0.243	0.496	0.427	0.489
8. EtOH/KOAc	0.250	0.470	0.430	0.505
9. EtOH/NaOAc	0.240	0.460	0.424	0.494

References

1. Hayduk, W.; Laudie, H. Prediction of Diffusion Coefficients for Nonelectrolytes in Dilute Aqueous Solutions. *Aiche J* **1974**, *20*, 611–615, doi:10.1002/aic.690200329.

2. Hao, L.; Leaist, D.G. Binary Mutual Diffusion Coefficients of Aqueous Alcohols. Methanol to 1-Heptanol. *J Chem Eng Data* **1996**, *41*, 210–213, doi:10.1021/je950222q.

3. Vany´sek, P. Ionic Conductivity and Diffusion at Infinite Dilution. *CRC Handbook of Chemistry and Physics, 93rd Edition* **2012**.

4. Hills, E.E.; Abraham, M.H.; Hersey, A.; Bevan, C.D. Diffusion Coefficients in Ethanol and in Water at 298K: Linear Free Energy Relationships. *Fluid Phase Equilibr* **2011**, *303*, 45–55, doi:10.1016/j.fluid.2011.01.002.
